# Breast carcinoma metastasis to the cheek: a case report

**DOI:** 10.1186/s13256-022-03326-6

**Published:** 2022-03-18

**Authors:** Ryo Miyazaki, Natsuko Kimoto, Satsuki Okamoto, Asami Tsuji, Yusuke Nishigushi, Tomoya Miyahara, Nozomu Takahashi, Tetsuji Omata

**Affiliations:** 1 Department of Oral and Maxillofacial Surgery, Kinan Hospital, 46-70 Shinjo-cho, Tanabe-shi, Wakayama 646-8588 Japan; 2 Department of Oral and Maxillofacial Surgery, Faculty of Medicine, Kagawa University, 1750-1 Ikenobe, Miki-cho, Kita-gun, Kagawa 761-0793 Japan; 3 Graduate School of Dentistry, Second Department of Oral and Maxillofacial Surgery, Osaka Dental University, 8-1 Kuzuhahanazono-cho, Hirakata-shi, Osaka 573-1121 Japan; 4 Department of Oral and Maxillofacial Surgery, Faculty of Life Sciences, Kumamoto University, 1-1-1 Honjo, Chuo-ku, Kumamoto-shi, Kumamoto 860-8556 Japan

**Keywords:** Breast cancer, Metastasis, Buccal, Oral

## Abstract

**Background:**

Breast carcinoma is a common tumor in women, but it rarely metastasizes to the oral region. Furthermore, metastases to the oral region occur mainly to the maxillary and mandibular bone and rarely to soft tissue.

**Case presentation:**

We describe a case of breast cancer metastasis to the buccal area. Examination of the right buccal mass of a 66-year-old Japanese woman was suggestive of breast cancer metastasis, and a breast lump was detected. Since receiving hormone-based treatment, the patient has survived more than 5 years and is now in remission.

**Conclusions:**

An oral metastatic lesion may be the first sign of breast carcinoma; oral surgeons should be aware of this possibility.

## Background

Metastasis to the oral area is rare, accounting for approximately 1% of all malignant tumors in the oral cavity [1]. Breast cancer is one of the most frequent malignant tumors in women and is known to frequently metastasize to the bone. It is reported that 3.6% of all breast cancers develop bone metastases [2]. While breast cancer as primary tumor is the most common source of metastasis to the oral cavity in women, metastasis to the buccal area is uncommon [3]. Its rarity occasionally makes its diagnosis difficult for both clinicians and pathologists. However, the oral cavity is highly visible, and discoveries of metastasis to the oral region may be an important clue in the detection of primary cancer.

Herein, we report a case in which breast cancer metastasis to the right cheek was observed before the primary tumor was identified.

## Case report

**Fig. 1:**
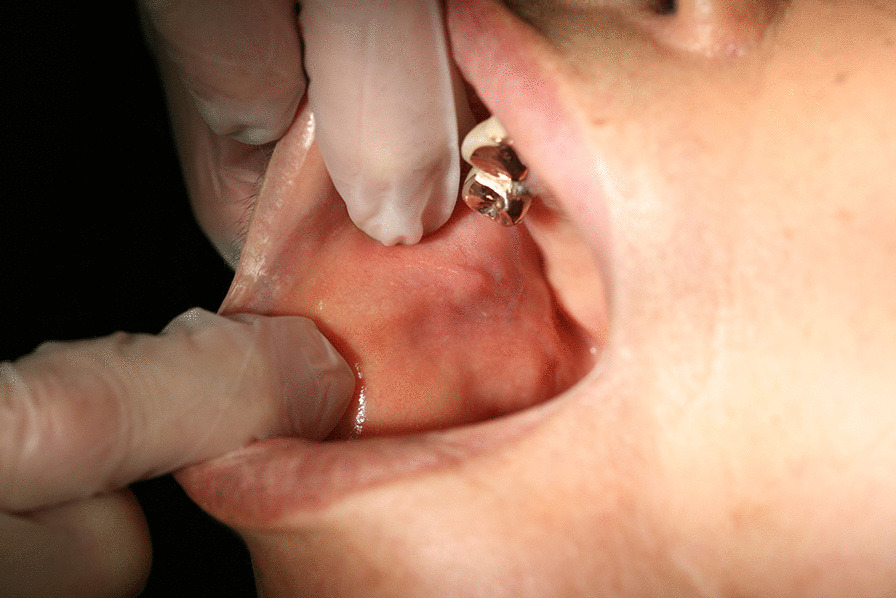
Intra-oral photo taken at the patient’s first visit, demonstrating buccal hard swelling with normal mucosa

**Fig. 2:**
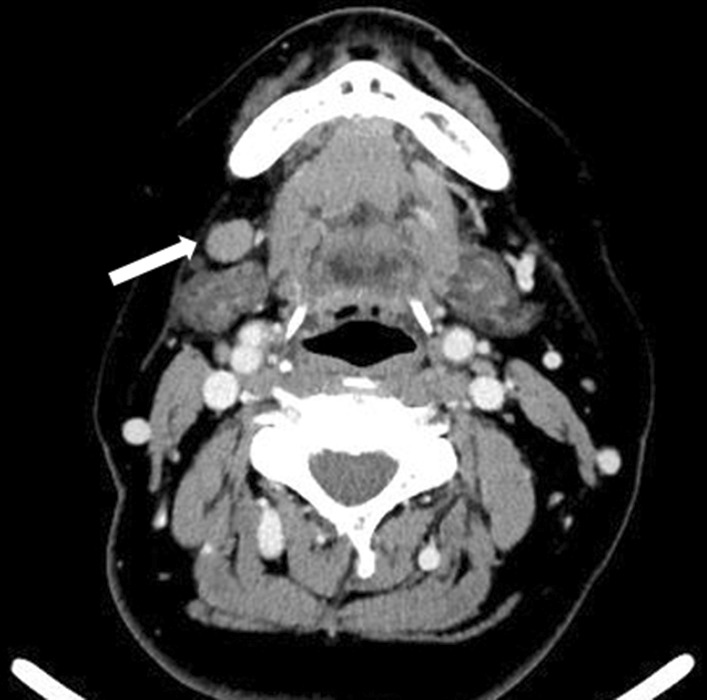
Contrast-enhanced computed tomography showing swelling of the right submandibular lymph node (arrow)

**Fig. 3:**
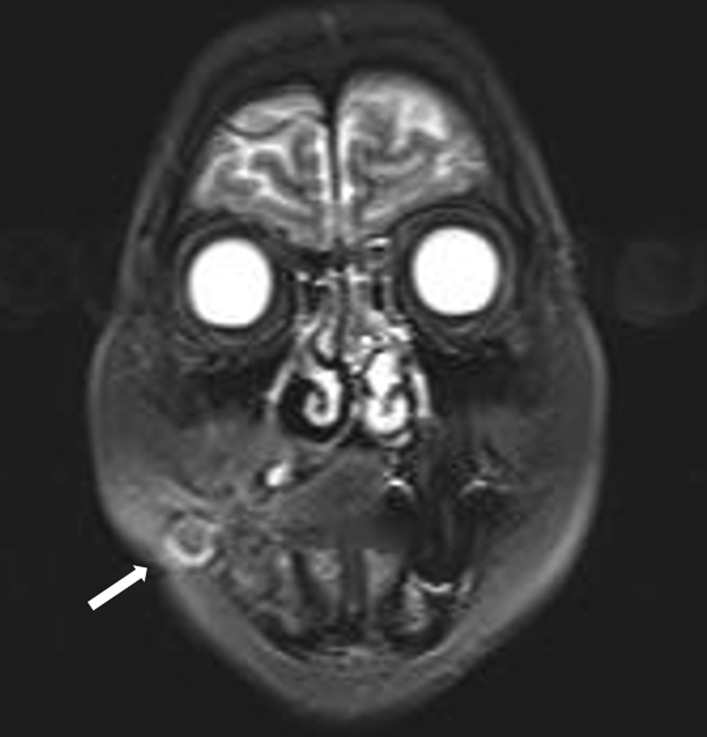
T2 fat-suppression magnetic resonance imaging demonstrating a mass in the right buccal region (arrow)

**Fig. 4:**
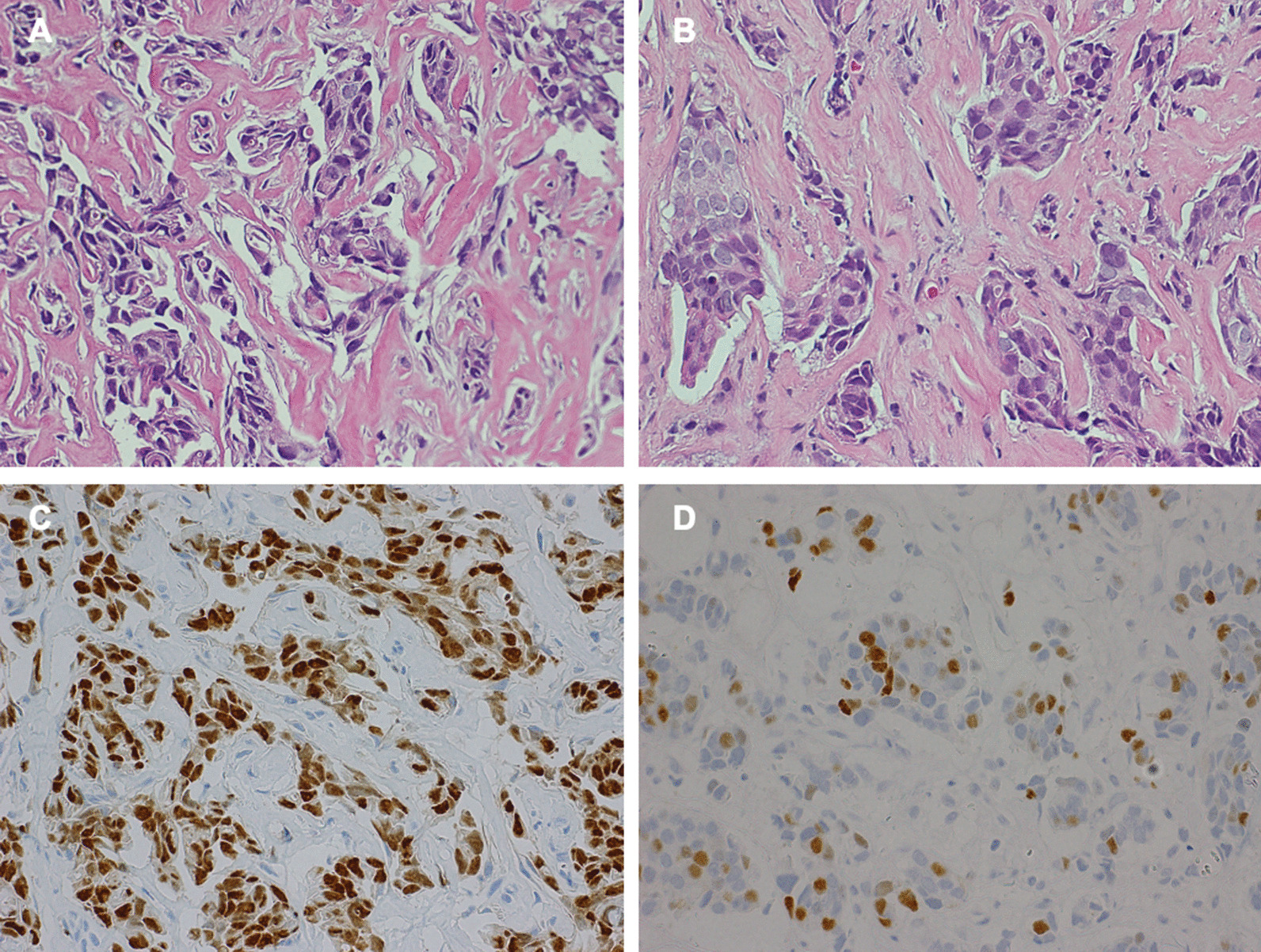
Hematoxylin–eosin staining revealing the large and small alveolar structures of tumor cells with nuclei of different sizes. **A** Buccal, ×400. **B** Breast, ×400. Immunohistochemical staining of the buccal lesion showing that the percentage of cells staining positive for estrogen receptor (ER) (**C**, ×400) and progesterone receptor (PgR) (**D**, ×400) was > 90% and 1–10%, respectively

In 2016, a 66-year-old Japanese woman was referred to our hospital with a chief complaint of right buccal swelling that had begun 1 month earlier. A hard, mobile mass was detected under her right buccal mucosa (Fig. 1). The mass caused her no pain, and her facial appearance was normal. Computed tomography demonstrated a clearly demarcated lesion under the right buccal mucosa along with swollen submandibular lymph nodes (Fig. 2). The buccal mass showed a low signal on T1- and T2-weighted magnetic resonance imaging and a high signal on T2 fat-suppression imaging (Fig. 3). Biopsy of the buccal mass was performed, and pathology was indicative of breast carcinoma metastasis (Fig. 4A).

**Fig. 5:**
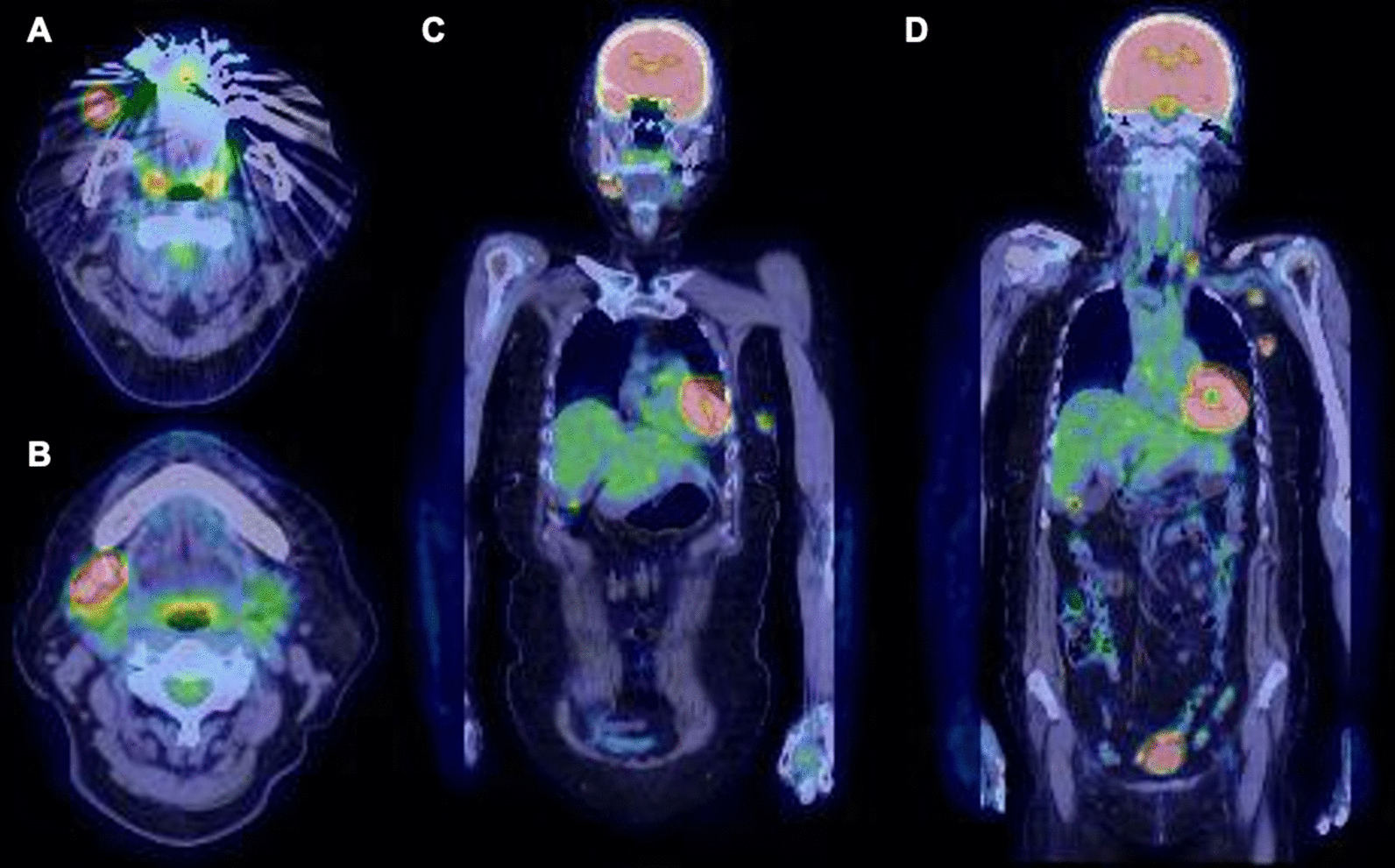
FDG-PET/CT showed high signals in the right buccal region (**A**), right submandibular lymph node (**B**), left breast (**C**), and lymph nodes in the left supraclavicular fossa and axilla (**D**)

One month before her visit, concurrent to the discovery of the buccal mass, she also noticed a left breast lump. After biopsy of the buccal mass, ultrasonography revealed an 18 × 17 × 22 mm mass located in the upper left breast, and a core needle biopsy performed by a breast surgeon resulted in the diagnosis of breast carcinoma as well as scirrhous carcinoma (Fig. 4B). Fluorodeoxyglucose (FDG) positron emission tomography–computed tomography (PET/CT) showed FDG avidity in the patient’s right cheek, submandibular area, left breast, supraclavicular fossa, and axilla (Fig. 5). Immunohistochemistry revealed tumor cells positive for estrogen receptor (ER) and progesterone receptor (PgR) and no amplification of the *HER2* gene (Fig. 4C, D).

After consultation with the breast surgeon, as the patient was postmenopausal, anastrozole (ANA) was used as hormonal treatment. Seven months later, no palpable mass was found in the oral and neck region, and FDG-PET/CT demonstrated partial response of the primary breast lesion and complete response of the buccal and submandibular metastases. In 2018, CT showed swelling of the right submandibular lymph nodes; at that point, treatment with fulvestrant was initiated, and it proved effective. However, the right submandibular painful lymphadenopathy reappeared in 2020. CT demonstrated swelling of the right cheek and bilateral neck lesions, and a regimen of the cyclin-dependent kinase inhibitor abemaciclib with fulvestrant was prescribed and has been used to this day. As of August 2021, the oral and neck lesions have disappeared and the chest lesions are well controlled.

## Discussion

Most malignant tumors arising in buccal space are salivary grand tumors, such as adenoid cystic carcinoma, acinic cell carcinoma, and mucoepidermoid carcinoma [4]. Therefore, metastasis of a tumor to the oral cavity is uncommon and challenging for oral surgeons and pathologists. The tumors metastatic to the oral region differ by gender. The most common primary sites in women are the breast, genitourinary or gynecologic sites, kidneys, and colorectum; in males, the lung, kidneys, liver, and prostate are predominant [5, 6]. In Japan, choriocarcinoma is reported to be the most common cancer metastasizing to the mouth/jaw among women [7], and 0.02–25% of oral metastases are breast carcinoma [5, 8, 9].

Oral cavity metastasis is mostly to the jaw, mandible, or maxilla, and far more cases of metastatic lesions in the jawbone rather than soft tissue have been reported, despite jawbone metastases being more difficult to detect because of their relative invisibility [6]. In oral soft tissues, the gingiva is the most frequent site [6]. It has been speculated that inflammation such as periodontitis may contribute to the attraction of metastatic cells.

Metastasis can occur through direct extension (permeation), through lymphatics or blood vessels (embolic spread), or by transplantation [10]. In our patient’s case, her cancer spread to nodes in submandibular, supraclavicular, and axillary sites; hence, we suspect that the tumor metastasized through the lymphatic vessels.

Breast cancer is the most common cancer among women in Japan and frequently metastasizes to the bones. In the oral region, the most common metastatic site of breast carcinoma is the jaw; the oral soft tissues are rarely affected [8]. To properly determine the treatment strategy for metastatic breast cancer, evaluation of the patient’s menopause status and the expression of ER, PgR, and HER2 is essential. Overexpression of HER2 is associated with a worse clinical course and a worse survival rate [11]. In addition, hormone therapy depends on ER and PgR status; therefore, ER and PgR expressions are key factors in the prognosis. Our patient’s first diagnosis was stage IV breast carcinoma. Biopsy findings and hormone receptor status being ER- and PgR-positive and HER2-negative guided our choices for hormonal therapy.

Metastatic tumors in the oral and maxillofacial region manifest various clinical and radiographic features. Soft-tissue metastases are often misjudged as benign tumors such as pyogenic granuloma, hemangioma, giant cell granuloma, and irritation fibroma [12]. According to a review of 114 cases of metastatic jaw tumors, the most common jaw symptom in such patients is pain [13]. Other symptoms such as swelling, the presence of an intraoral mass, loose or extruded teeth, cortical expansion, regional lymphadenopathy, gum irritation, ulceration, exophytic growth, halitosis, numbness or paresthesia of the lower lip, and trismus might be clues [14]. “Numb chin” syndrome is one of the symptoms raising suspicion of a metastatic process [15]. Occasionally, patients present with nonspecific clinical features; therefore, a thorough medical interview of patients with a known malignancy is essential. However, in 25% of cases of metastatic tumors in the oral and maxillofacial region, oral metastases were found to be the first sign of the metastatic spread. Furthermore, in 23% of oral metastasis cases, the oral lesion was the first indication of an undiscovered malignancy at a distant site [6]. Therefore, in cases in which one or more lesions with uncommon histological features are identified, a whole-body search may be necessary. It was reported that most patients die within 1 year of the discovery of oral metastasis, and the estimated 4-year survival rate is 10% [16]. Early detection and diagnosis of oral metastasis is thus extremely important. Fortunately, diagnostic and therapeutic technologies for this purpose have evolved dramatically in recent years.

## Conclusion

This case report highlights an extremely rare case of breast cancer metastasizing to the cheek. Dental professionals should be aware that oral lesions they encounter may be linked to an as-yet undiscovered malignancy at a distant site.

## Data Availability

Not applicable.
